# Hydrothermal Synthesis of Co-Exposed-Faceted WO_3_ Nanocrystals with Enhanced Photocatalytic Performance

**DOI:** 10.3390/nano12162879

**Published:** 2022-08-22

**Authors:** Xianjun Niu, Yien Du, Jing He, Xiaodong Li, Guangming Wen

**Affiliations:** 1 Department of Chemistry and Chemical Engineering, Jinzhong University, Jinzhong 030619, China; 2 Department of Scientific Research, Jinzhong University, Jinzhong 030619, China

**Keywords:** tungsten trioxide, co-exposed crystal facets, photocatalytic activity, synergistic effect

## Abstract

In this paper, rod-shaped, cuboid-shaped, and irregular WO_3_ nanocrystals with different co-exposed crystal facets were prepared for the first time by a simple hydrothermal treatment of tungstic acid colloidal suspension with desired pH values. The crystal structure, morphology, specific surface area, pore size distribution, chemical composition, electronic states of the elements, optical properties, and charge migration behavior of as-obtained WO_3_ products were characterized by powder X-ray diffraction (XRD), field emission scanning electron microscopy (FESEM), transmission electron microscopy (TEM), high-resolution transmission electron microscopy (HRTEM), X-ray photoelectron spectroscopy (XPS), fully automatic specific surface area and porosity analyzer, UV–vis absorption spectra, photoluminescence (PL) spectra, and electrochemical impedance spectroscopy (EIS). The photocatalytic performances of the synthesized pH*x*-WO_3_ nanocrystals (*x* = 0.0, 1.5, 3.0, 5.0, and 7.0) were evaluated and compared with the commercial WO_3_ (CM-WO_3_) nanocrystals. The pH7.0-WO_3_ nanocrystals with co-exposed {202} and {020} facets exhibited highest photocatalytic activity for the degradation of methylene blue solution, which can be attributed to the synergistic effects of the largest specific surface area, the weakest luminescence peak intensity and the smallest arc radius diameter.

## 1. Introduction

Transition metal oxide nanocrystals with tailored shapes and reactive facets have sparked intense research interest over the past two decades due to their many intrinsic morphology and crystal plane-dependent properties [1]. Among many transition metal oxides, tungsten trioxide (WO_3_) is a typical narrow band gap (2.4~2.8 eV) *n*-type 5d^0^ transition metal oxide semiconductor, which plays a key role in many applications such as gas sensors, photoelectrochemical water splitting, electrochromic and photochromic devices, and photocatalytic systems [2,3]. Generally, WO_3_ nanocrystals are formed by sharing the corners and edges of WO_6_ octahedra, which exists in five polymorphs namely *ε*-WO_3_ (monoclinic II, space group *Pc*, stable temperature <−43 °C), *δ*-WO_3_ (triclinic, space group *P*$\overset{-}{1}$, stable temperature −43~17 °C), *γ*-WO_3_ (monoclinic I, space group *P*2_1_/*n*, 17~330 °C), *β*-WO_3_ (orthorhombic, space group P*mnb*, transition temperature 330~740 °C), and *α*-WO_3_ (tetragonal, space group *P*4/*nmm*, stable temperature > 740 °C) [4,5]. Among the five different crystalline phases of WO_3_, the *γ*-WO_3_ is the most thermodynamically stable phase at room temperature [5]. Therefore, the commonly mentioned WO_3_ refers specifically to *γ*-WO_3_. WO_3_ has been considered a promising visible-light-driven photocatalyst not only due to its high hole mobility and moderate hole diffusion length, but also due to its photosensitivity, inherently good electron transport properties, resistibility to photocorrosion, and low cost [6,7]. However, the low conduction band level of WO_3_ inhibits its ability to react with electron acceptors and increases the recombination of photogenerated electron–hole pairs, resulting in poor photocatalytic activity for the degradation of organic pollutants [8,9]. Therefore, strenuous efforts have been made to improve the photocatalytic activity of WO_3_ materials, such as controlling the particle size, crystal structure, crystal morphology, crystal surface exposure, crystal composition, etc. [10,11,12]. In particular, the morphology and exposed crystal surface of WO_3_ material have an important influence on photocatalytic performance. In view of this, extensive research work has been carried out to synthesize numerous morphological WO_3_ materials with specific exposed crystal surfaces via different methods. For instance, Xie et al. [13] synthesized a quasicubic-like monoclinic WO_3_ crystal with co-exposed {002}, {200} and {020} facets, and a rectangular sheet-like monoclinic WO_3_ crystal with predominant {002} facet via a simple solvothermal synthesis method. Han et al. [14] synthesized monodisperse triclinic WO_3_ nanoparticles with co-exposed {001}, {100} and {010} facets via a simple hydrothermal method. D’Arienzo et al. [15] synthesized WO_3_ nanocrystals with tailored morphology (rectangular nanocrystals, square-like platelets, and rectangular platelets) and definite prominent surfaces (co-exposed high-energy {020} and {002} facets) via a simple hydrothermal synthesis method. Dirany et al. [16] synthesized a well-crystallized orthorhombic quadrangular WO_3_ nanoplates with dominant exposed {020} facets via free template aqueous mineralization processes. Bu et al. [17] fabricated a well-defined hierarchical WO_3_ nanoflower-like thin film photoanode composed of WO_3_ nanoflakes with mismatched {002} and {020} facets exposed via a complex template assistant method.

In this study, rod-shaped WO_3_ nanocrystals with co-exposed {002}, {020} and {200} facets and growing along the [002] direction (pH0.0-WO_3_, pH1.5-WO_3_, and pH3.0-WO_3_), cuboid-shaped WO_3_ nanocrystals with co-exposed {020} and {200} facets (pH1.5-WO_3_ and pH3.0-WO_3_), irregular WO_3_ nanocrystals with co-exposed {110} and {002} facets (pH5.0-WO_3_) and {202) and {020} crystal facets (pH7.0-WO_3_), were successfully synthesized by using the exfoliated white tungstic acid colloidal suspension as precursor via a mild hydrothermal method. A series of techniques were used to characterize the crystal structure, morphology, macrostructure and analyze the chemical composition, electronic states of the elements, optical properties, and charge migration behavior of as-obtained WO_3_ products. The photocatalytic activity of the as-prepared pH*x*-WO_3_ nanocrystals was investigated. Compared to CM-WO_3_ nanocrystals, the irregular pH7.0-WO_3_ nanocrystals with co-exposed {202} and {020} crystal facets showed higher photocatalytic activity.

## 2. Materials and Methods

### 2.1. Materials

Sodium tungstate dihydrate (Na_2_WO_4_·2H_2_O, 99.5%), nitric acid (HNO_3_, 65–68%), and tetramethylammonium hydroxide (TMAOH, 96%) were purchased from Shanghai Macklin Biochemical Co., Ltd. (Shanghai, China), Damao Chemical Reagent Factory (Tianjin, China), and Dubai Biological Technology Co., Ltd. (Shanghai, China), respectively. The above three chemical reagents were used as received without further purification.

### 2.2. Synthesis of WO_3_ Nanocrystals

The WO_3_ nanocrystals were synthesized by a mild hydrothermal method. Briefly, 20.0 g of the white Na_2_WO_4_·2H_2_O powders were dissolved in 2.0 L of 1.0 mol/L HNO_3_ with stirring for 3 days at room temperature, while the HNO_3_ solution was replaced daily with a fresh solution of equal volume and equal concentration, to prepare the white tungstic acid monohydrate (H_2_WO_4_·H_2_O) powders. 14.0 g of H_2_WO_4_·H_2_O and 18.2 g of TMAOH were dissolved in 140 mL of deionized water with stirring for 15 min and then transferred into two 100 mL of Teflon-lined autoclave on average. After adequate sealing, the two autoclaves were fixed in a homogeneous reactor and heated at 85 °C for 24 h with constant stirring to prepare a TMA^+^-intercalated tungstic acid compound and then cooled to ambient temperature. The above compound was dispersed in 500 mL of deionized water and stirred at room temperature for 3 days to obtain a white tungstic acid colloidal suspension. An amount of 65 mL of the precursor colloidal suspension was transferred into a 100 mL Teflon-lined autoclave, and then adjusted to the set pH value (pH = 0.0, 1.5, 3.0, 5.0, and 7.0) under stirring conditions. After the autoclaves were tightly sealed, they were put into the constant temperature blast drying oven for a reaction at 180 °C for 24 h. Yellow WO_3_ products (pH0.0-WO_3_, pH1.5-WO_3_, pH3.0-WO_3_, pH5.0-WO_3_, and pH7.0-WO_3_) were obtained by centrifuge after multiple times washing with deionized water and drying at room temperature for longer than 24 h, and then calcined in a high-temperature box furnace at 500 °C for 4 h.

### 2.3. Sample Characterization

Powder X-ray diffraction analysis was carried out using an XRD-6100 (Shimadzu, Kyoto, Japan) with monochromated Cu Kα radiation (λ = 0.15406 nm). The morphology of the precursor Na_2_WO_4_·2H_2_O, H_2_WO_4_·H_2_O, and the synthesized WO_3_ samples were observed using a field emission scanning electron microscopy (FESEM, Hitachi SU8100, Tokyo, Japan). Transmission electron microscopy (TEM) and high-resolution transmission electron microscopy (HRTEM) images were obtained by using an FEI TALO F200S (Portland, OR, USA) at an operating voltage of 200 kV. X-ray photoelectron spectroscopy (XPS) was performed on a K-Alpha instrument (Thermo Fisher Scientific, New York, NY, USA) and calibrated by the binding energy of C 1s 284.6 eV. The Brunauer–Emmett–Teller surface areas were obtained by using a micromeritics ASAP 2020 nitrogen adsorption instrument (Micromeritics Instrument Corp., Atlanta, GA, USA) at 77 K. Ultraviolet–visible (UV–Vis) absorption spectra of the WO_3_ samples were recorded with a UV–Vis spectrophotometer (UV-2600, Shimadzu, Kyoto, Japan). Photoluminescence analysis of the WO_3_ samples was measured on a fluorescence spectrometer (PL, HORIBA Fluoromax-4, HORIBA Instruments Inc., Kyoto, Japan) and the emission spectrum were excited at a wavelength of 325 nm. The electrical measurements were performed using electrochemical impedance spectroscopy (EIS, CHI600E, Shanghai Chenhua Instrument Co., Ltd., Shanghai, China) with indium-tin oxide glass, platinum plate (opening area: 1 cm^2^), and Ag/AgCl (saturated KCl solution) as the working electrode, counter electrode, and reference electrode, respectively, under the irradiation of a 300 W xenon lamp. The EIS were performed in a 0.2 mol/L Na_2_SO_4_ solution with a frequency range from 0.01 Hz to 100 kHz under open circuit potential conditions. The working electrode was prepared by a simple method as follows: 25 mg of WO_3_ was dispersed in 1 mL of 5% polyvinylidene fluoride (PVDF) solution and stirred for 60 min to form uniform WO_3_ slurry. Then, 20 μL of the slurry was dripped on the ITO glass with a 3 cm × 1 cm area and dried at 80 °C for 12 h.

### 2.4. Photocatalytic Experiments

Photocatalytic activities of the synthesized pH*x*-WO_3_ samples were evaluated for the decolorization of methylene blue (MB) aqueous solution with a 175 W low-pressure mercury lamp at ambient temperature. Typically, 150 mg of the WO_3_ sample (pH0.0-WO_3_, pH1.5-WO_3_, pH3.0-WO_3_, pH5.0-WO_3_, pH7.0-WO_3_, and CM-WO_3_) was dispersed in 150 mL of 15 mg/L MB aqueous solution (4.07 × 10^−5^ mol/L). Before the sample was exposed to ultraviolet–visible light irradiation, the suspension was magnetically stirred in the dark for 2 h to ensure the adsorption–desorption equilibrium of the MB dye on the surfaces of WO_3_ sample. Then, at given time of irradiation, 3 mL of suspension was taken out and the WO_3_ sample was immediately centrifuged to analyze the supernatant liquor by an ultraviolet-visible spectrophotometer (TU 1901, Beijing Purkinje General Instrument Co., Ltd., Beijing, China).

## 3. Results

### 3.1. XRD Analysis

**Figure 1:**
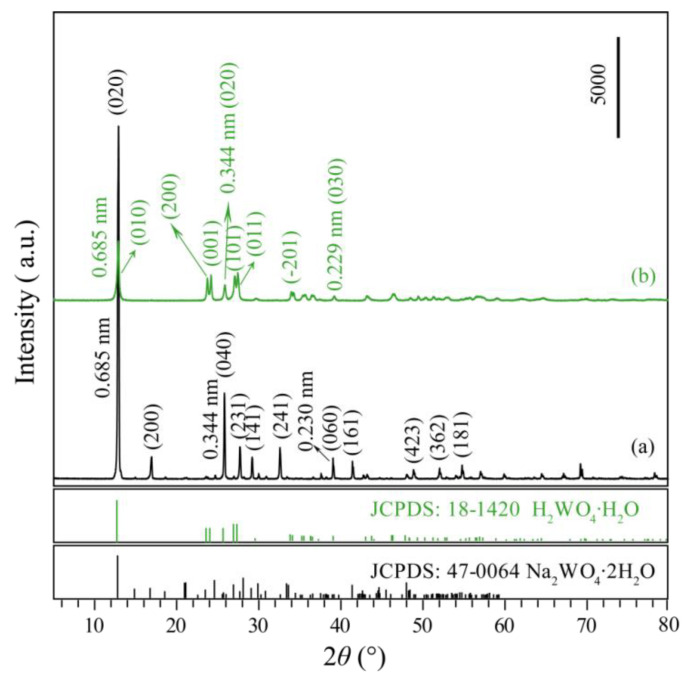
XRD patterns of (**a**) commercial Na_2_WO_4_·2H_2_O precursor and (**b**) prepared H_2_WO_4_·H_2_O sample.

The commercial precursor, orthorhombic Na_2_WO_4_·2H_2_O, is confirmed by the powder X-ray diffraction (XRD) pattern (JCPDS no. 47-0064, *a* = 10.592, *b* = 13.858, and *c* = 8.479), as shown in Figure 1a. The existence of the main diffraction peaks of (020), (040), and (060), and the corresponding *d* values are 0.685, 0.344, and 0.229 nm, respectively, indicate that the precursor Na_2_WO_4_·2H_2_O belongs to layered compounds. After the precursor Na_2_WO_4_·2H_2_O was treated with 1.0 mol/L HNO_3_ for 3 days at room temperature, monoclinic H_2_WO_4_·H_2_O (JCPDS no. 18-1420, *a* = 0.750, *b* = 0.693, *c* = 0.370 nm, and *β* = 90.5°) were obtained (Figure 1b). The diffraction peaks of H_2_WO_4_·H_2_O at 2*θ* = 12.92°, 25.90°, and 39.28° correspond to the (010), (020), and (030) crystal planes and the crystal plane spacing is 0.685, 0.344, and 0.229 nm, respectively, indicating that the H_2_WO_4_·H_2_O sample also has a layered structure.

**Figure 2:**
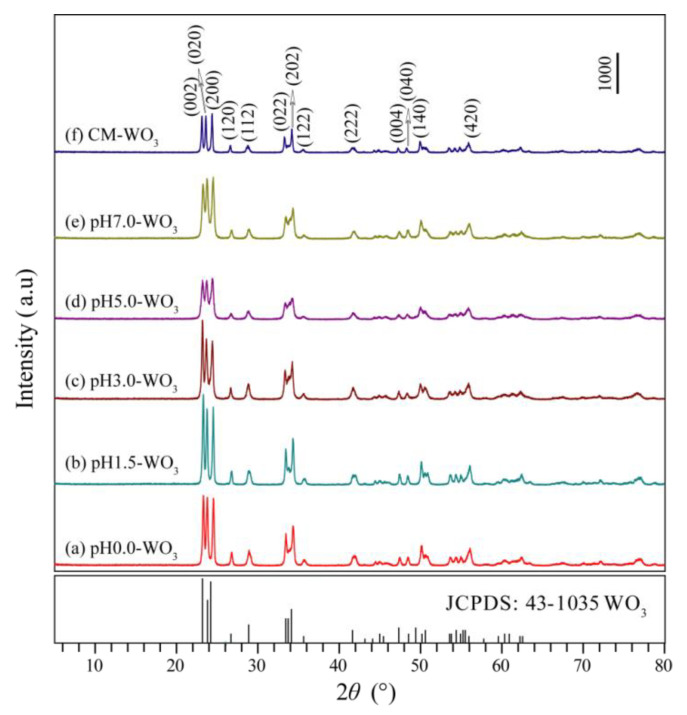
XRD patterns of WO_3_ samples synthesized by hydrothermal method using tungstic acid colloidal suspension with different pH values (0.0–7.0): (**a**) pH0.0-WO_3_, (**b**) pH1.5-WO_3_, (**c**) pH3.0-WO_3_, (**d**) pH5.0-WO_3_, (**e**) pH7.0-WO_3_, and (**f**) the commercial WO_3_ (CM-WO_3_) sample.

Figure 2 shows the XRD patterns of WO_3_ samples synthesized by hydrothermal treatment tungstic acid colloidal suspension with different pH values (0.0–7.0) and the commercial WO_3_ (CM-WO_3_) sample. The diffraction peaks of the pH0.0-WO_3_, pH1.5-WO_3_, pH3.0-WO_3_, pH5.0-WO_3_, pH7.0-WO_3_, and CM-WO_3_ samples at 2*θ* values of 23.32°, 23.80°, 24.54°, 26.78°, 28.92°, 33.46°, 34.34°, 35.64°, 41.92°, 47.44°, 48.48°, 50.12°, and 56.10° can be indexed to (002), (020), (200), (120), (112), (022), (202), (122), (222), (004), (040), (140), and (420) planes of the monoclinic structure of WO_3_ (space group: *P*2_1_/*n*(14)) with the standard card JCPDS no. 43-1035, indicating that the samples have high crystallographic purity. The strong and sharp reflection peaks and very horizontal baselines in Figure 2a–e indicate the high crystallinity and purity in the as-synthesized WO_3_ powder samples [3]. The relative intensities of the (002), (020), and (200) diffraction peaks of the as-synthesized WO_3_ powder samples are different, indicating that different controlling agents may adjust the different exposed facets [2].

### 3.2. FESEM and TEM Analysis

**Figure 3:**
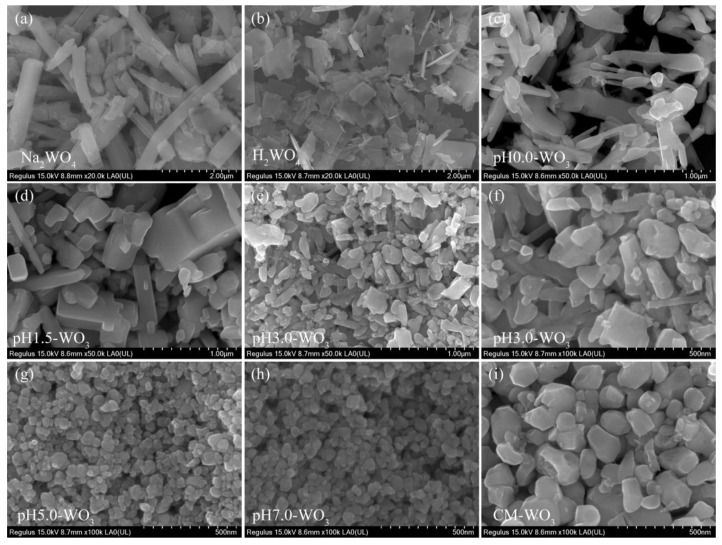
FESEM images for: (**a**) Na_2_WO_4_, (**b**) H_2_WO_4_, (**c**) pH0.0-WO_3_, (**d**) pH1.5-WO_3_, (**e**,**f**) pH3.0-WO_3_, (**g**) pH5.0-WO_3_, (**h**) pH7.0-WO_3_, and (**i**) CM-WO_3_.

The morphology of the Na_2_WO_4_, H_2_WO_4_, and the as-synthesized pH*x*-WO_3_ samples were characterized by FESEM. Figure 3a illustrates a typical FESEM image of Na_2_WO_4_ sample. It can be seen that most of the samples exhibit square rod-like morphology with wide size distributions (length ranges from hundreds of nanometers to several micrometers and width ranges from 0.1 to 0.5 µm). After the ion exchange reaction, the obtained H_2_WO_4_ sample exhibits irregular rod-like morphology with wide size distributions (length ranges from 0.1 to 1.5 µm and width ranges from 0.1 to 0.5 µm) (Figure 3b), which is probably caused by the fracture of the rod-like Na_2_WO_4_ particles under the condition of intense agitation. Figure 3c shows a typical FESEM image of pH0.0-WO_3_ product prepared by hydrothermal treatment of TMA^+^-intercalated tungstic acid colloidal suspension in pH value of 0.0. Most of the products exhibit irregular rod-like morphology with wide size distributions (length is from 0.1 to 1.5 µm and width is from 30 to 230 nm). Figure 3d shows that when the pH value of colloidal suspension is 1.5, the pH1.5-WO_3_ product exhibits a discordant rod-like shape with a length of about 0.2~2.2 µm and a width of about 50~30 nm, and several irregular particles. Some discordant rod-like nanoparticles with 70~350 nm in length and 30~70 nm in width and many irregular nanoparticles with wide size distributions are observed when the pH value of colloidal suspension is 3.0, as shown in Figure 3e,f. As shown in Figure 3g,h, the pH5.0-WO_3_ and pH7.0-WO_3_ samples consist of large amounts of cuboid, spherical and irregular nanoparticles with an average size of 34.8 nm and 37.9 nm, respectively. CM-WO_3_ sample consists of large amounts of irregular nanoparticles with an average size of 137 nm, as shown in Figure 3i.

**Figure 4:**
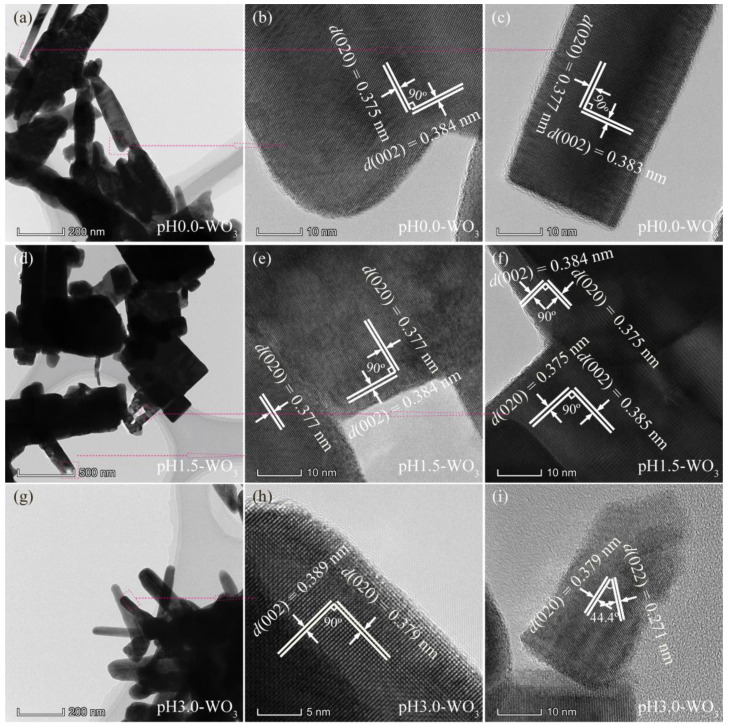
TEM and HRTEM images for: (**a**–**c**) pH0.0-WO_3_, (**d**–**f**) pH1.5-WO_3_, and (**g**–**i**) pH3.0-WO_3_.

**Figure 5:**
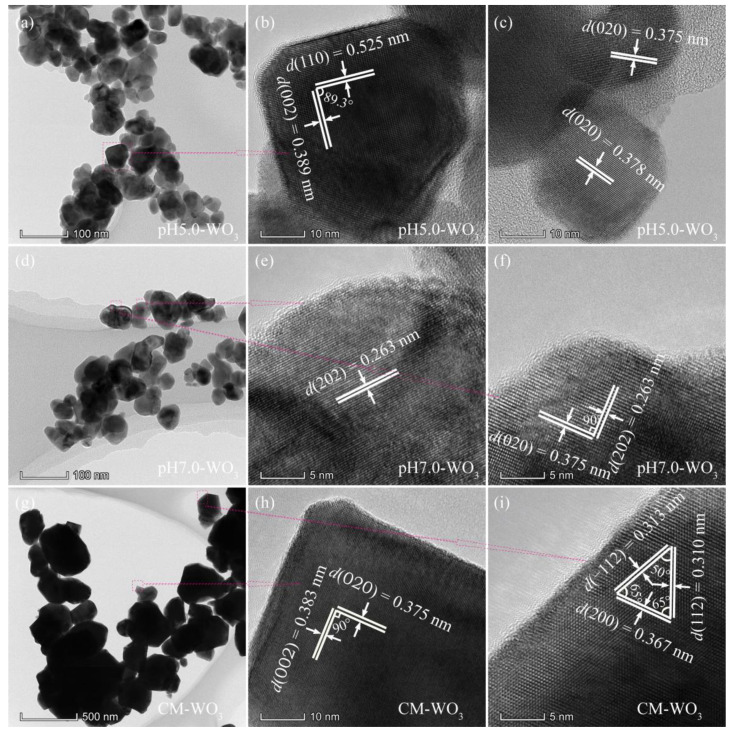
TEM and HRTEM images for: (**a**–**c**) pH5.0-WO_3_, (**d**–**f**) pH7.0-WO_3_, and (**g**–**i**) CM-WO_3_.

The structure of the as-synthesized pH*x*-WO_3_ samples was characterized by TEM, as shown in Figure 4 and Figure 5. Figure 4a shows the TEM image of the prepared pH0.0-WO_3_ crystals derived from the hydrothermal treatment of the TMA^+^-intercalated tungstic acid colloidal suspension at 180 °C with a reaction time of 24 h. The rod-shaped nanocrystals with a length of 200–700 nm and a width of 30–130 nm were observed. Figure 4b,c shows the corresponding HRTEM image of an individual pH0.0-WO_3_ nanorod taken from the marked area of the TEM images, revealing that the nanorod possesses the single-crystal structure. The lattice spacing of around 0.375 and 0.384 nm (or 0.377 and 0.383 nm) correspond the *d* spacing of WO_3_ (020) and (002) crystal planes, and the interfacial angle of 90° between them matches well with the theoretical value. Furthermore, the WO_3_ (020) and (002) crystal planes parallel to the sides and tops of the nanorod, respectively, and the longitudinal axis direction corresponds to the (002) crystal planes of WO_3_, indicating that the co-exposed facets of pH0.0-WO_3_ nanocrystals are {002}, {020} and {200} facets and the elongation of the nanorod is parallel to [002] direction. Rod-shaped WO_3_ crystals with a length of 0.3–1.5 µm and a width of 45–240 nm and cuboid-shaped WO_3_ crystals with a length of 250–800 nm were observed in the TEM image of pH1.5-WO_3_ crystals (Figure 4d). The corresponding HRTEM images (Figure 4e,f) showed the perpendicular (020) and (002) atomic planes of the rod-shaped and the cuboid-shaped WO_3_ crystals with a lattice spacing of 0.377 (or 0.375) and 0.384 (or 0.385) nm, respectively, and the (020) and (002) crystal planes parallel to the two sets of surfaces of rod-shaped (or cuboid-shaped) WO_3_ crystals, respectively, indicating that the co-exposed facets of pH1.5-WO_3_ crystals are {002}, {020} and {200} facets. Rod-shaped WO_3_ crystals with a length of 240–540 nm and a width of 40–110 nm were observed in the TEM image of pH3.0-WO_3_ crystals (Figure 4g). Figure 4h shows the HRTEM image of an individual pH3.0-WO_3_ nanorod taken from the marked area of the TEM images, revealing that the nanorod possesses a single-crystal structure and the lattice spacing of around 0.379 and 0.389 nm along the horizontal axis and longitudinal axis direction correspond to the *d* spacing of WO_3_ (020) and (002) crystal planes. The above analysis results further indicate that the co-exposed facets of pH3.0-WO_3_ nanocrystals are {002}, {020} and {200} facets, and the growth direction of the nanorod is along the [002] direction. The HRTEM image (Figure 4i) shows that the interplanar spacing of cuboid-shaped WO_3_ crystals are 0.379 and 0.271 nm with an interfacial angle of 44.4°, corresponding to the (200) and (022) planes of monoclinic WO_3_, respectively, which indicates that the corresponding set of side facets is {020} facets, and the predominant facets of the cuboid-shaped WO_3_ crystal is {200} crystal facets.

The pH5.0-WO_3_ sample consists of a large amount of irregular morphology nanocrystals with a size of 18~106 nm, as shown in Figure 5a. The corresponding HRTEM images (Figure 5b,c) show that the distances of the visible lattice fringes over a large area were measured to be 0.525, 0.389, and 0.375 (or 0.378) nm, which are in agreement with the lattice spacing of (110), (002) and (020) atomic planes of the monoclinic structure of WO_3_. In addition, the (110) and (002) crystal planes parallel to the sides of the irregular nanocrystal with an interfacial angle of 89.3°, indicating that the irregular nanocrystal with co-exposed of {110} and {002} facets on its sides, as shown in Figure 5b. Irregular-shaped WO_3_ nanocrystals with a size of 18–86 nm were observed in the TEM image of the pH7.0-WO_3_ sample, as shown in Figure 5d. The HRTEM images (Figure 5e,f) show that the distances of the visible lattice fringes over a large area were measured to be 0.263 and 0.375 nm, which are in agreement with the lattice spacing of (202) and (020) atomic planes of the monoclinic structure of WO_3_. Furthermore, the interfacial angle between (202) and (020) crystal planes is 90°, which is consistent with the theoretical value. Since the crystal planes perpendicular to (202) and (020) at the same time cannot be determined, there are no specific exposed crystal facets on the base surface of the pH7.0-WO_3_ nanocrystals with irregular morphology. The (202) and (020) crystal planes are parallel to the sides of the irregular nanocrystal, so the {202} and {020} crystal facets are co-exposed on the sides. Compressed hexagonal prismatic and irregular-shaped WO_3_ nanocrystals with a size of 55–730 nm were observed in the CM-WO_3_ sample, as shown in Figure 5g. The lattice spacings of 0.375 and 0.383 with an interfacial angle of 90° can be indexed to the (020) and (002) crystal planes, respectively (Figure 5h). Furthermore, the (020) and (002) crystal planes are parallel to the sides of the compressed hexagonal prism, indicating that the co-exposed crystal facets of the compressed hexagonal prism are {020}, {002} and {200} facets. The lattice spacing of 0.310, 0.313, and 0.367 with an interfacial angle of 50°, 65°, and 65° can be indexed to the (112), (−112), and (200) crystal planes, respectively (Figure 5i). Furthermore, the (−112) crystal plane parallels to the side of irregular nanocrystal, indicating that the side exposes {−112} crystal facets. Similar, since the crystal planes perpendicular to (112), (−112), and (200) at the same time cannot be determined, there is no specific crystal facets on the base surface of the CM-WO_3_ nanocrystals with irregular morphology.

### 3.3. Nitrogen Adsorption–Desorption Isotherms Analysis

**Figure 6:**
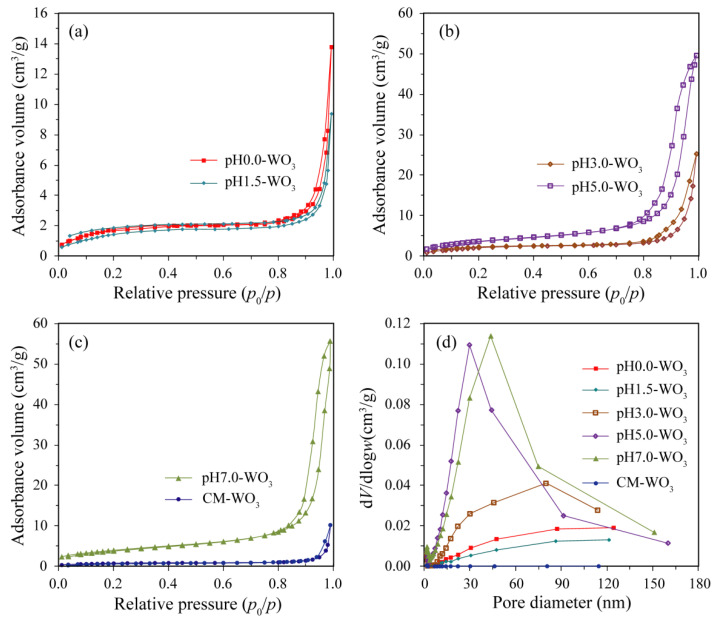
Nitrogen adsorption–desorption isotherm of (**a**) pH0.0-WO_3_ and pH1.5-WO_3_, (**b**) pH3.0-WO_3_ and pH5.0-WO_3_, (**c**) pH7.0-WO_3_ and CM-WO_3_, and (**d**) the corresponding the pore size distribution.

The Brunauer–Emmett–Teller (BET) surface area and pore size of the as-prepared pH*x*-WO_3_ (*x* = 0.0, 1.5, 3.0, 5.0, 7.0) and the commercial CM-WO_3_ samples were characterized using the nitrogen adsorption–desorption isotherms shown in Figure 6. It can be seen from Figure 6a–c, the adsorption isotherms of the as-prepared pH*x*-WO_3_ and CM-WO_3_ are type IV (BDDT classification) with type H3 hysteresis loops at relatively high pressure between 0.8 and 1.0, corresponding to slit-shaped pores, which are in accordance with the characteristic of nitrogen adsorption on macroporous absorbents [18]. Figure 6d shows the pore size distributions of the as-prepared pH*x*-WO_3_ and the commercial CM-WO_3_ samples. As shown in Figure 6d, there is no obvious pore size peak on the pore size distribution curves of pH0.0-WO_3_, pH1.5-WO_3,_ and CM-WO_3_, which indicates that these particles do not exhibit intrinsic porosity [19]. However, the pH3.0-WO_3_, pH5.0-WO_3_, and pH7.0-WO_3_ samples present a relatively wide range of pore size distribution from 7.0 to 113.8 nm, 3.5 to 160.0 nm, and 5.3 to151 nm, respectively [20]. The BET surface areas of the as-prepared pH0.0-WO_3_, pH1.5-WO_3_, pH3.0-WO_3_, pH5.0-WO_3_, pH7.0-WO_3_ as well as CM-WO_3_ are determined to be 6.4, 5.4, 7.7, 13.7, 14.3, and 2.6 m^2^/g, respectively. That is, the BET surface area decreases in the following order: pH7.0-WO_3_ > pH5.0-WO_3_ > pH3.0-WO_3_ > pH0.0-WO_3_ > pH1.5-WO_3_ > CM-WO_3_. The pH7.0-WO_3_ exhibits the largest BET surface area among the samples, being 5.50, 2.23, 2.65, 1.86, and 1.04 times higher than that of CM-WO_3_, pH0.0-WO_3_, pH1.5-WO_3_, pH3.0-WO_3_, and pH5.0-WO_3_, respectively. The increased BET surface areas of the WO_3_ sample can provide more adsorption and reaction sites for the organic dye in the photocatalytic process, which may be beneficial to improving the photocatalytic activity [21].

### 3.4. X-ray Photoelectron Spectroscopy Analysis

**Figure 7:**
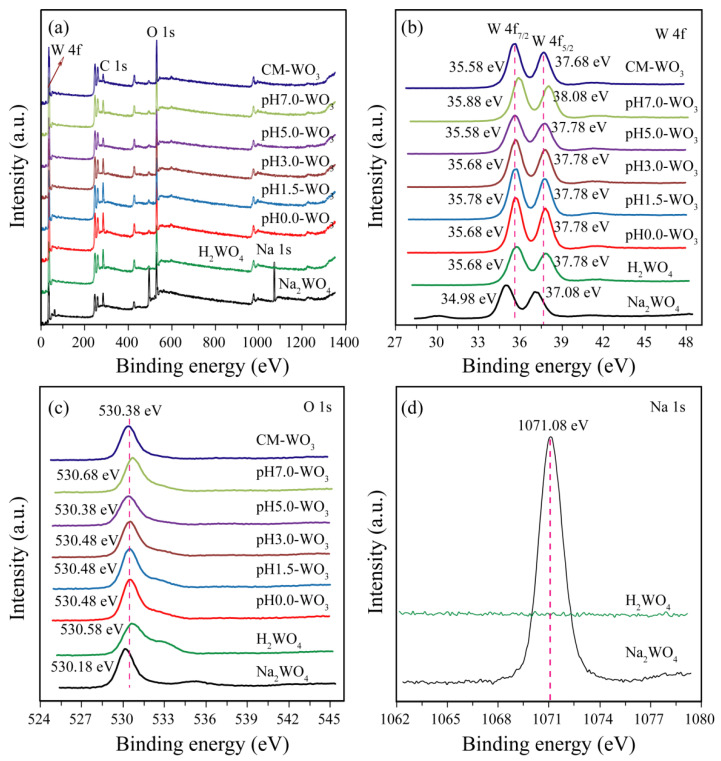
XPS spectra of Na_2_WO_4_, H_2_WO_4_, as-prepared pH*x*-WO_3,_ and the CM-WO_3_ samples: (**a**) survey scan; high-resolution (**b**) W 4f, (**c**) O 1s, and (**d**) Na 1s spectrum.

To complete the previous analysis, X-ray photoelectron spectroscopy (XPS) analysis reveals the surface chemical composition and electronic states of the elements of the Na_2_WO_4_, H_2_WO_4_, as-prepared pH*x*-WO_3_ and CM-WO_3_ samples (Figure 7). The C 1s peak at 284.88 eV observed in the survey scan is due to carbon contamination used to calibrate the binding energy [22]. The full wide-scan spectra of the Na_2_WO_4_, H_2_WO_4_, as-prepared pH*x*-WO_3,_ and CM-WO_3_ samples are presented in Figure 7a, from which we observe clearly characteristic peaks of Na (exists only in Na_2_WO_4_), W, O and C elements. The high-resolution W 4f spectrum in Figure 4b, displays two peaks with binding energy values of 37.08~38.08 and 34.98~35.88 eV for W 4f_5/2_ and W 4f_7/2_ indicating the W(VI) oxidation state of Na_2_WO_4_, H_2_WO_4_, as-prepared pH*x*-WO_3_ and CM-WO_3_ samples [23]. The O 1s peak (Figure 4c) at 530.18~530.68 eV matches well with oxygen species in the Na_2_WO_4_, H_2_WO_4_, as-prepared pH*x*-WO_3,_ and CM-WO_3_ samples, which can be assigned to typical surface lattice oxygen [23]. The Na 1s peak (Figure 4d) at 1071.08 eV matches well with sodium species in the Na_2_WO_4_ sample. No peak of Na 1s was observed in H_2_WO_4_, further indicating that Na^+^ ions in Na_2_WO_4_ were well displaced by H^+^ ions.

### 3.5. Optical Properties

**Figure 8:**
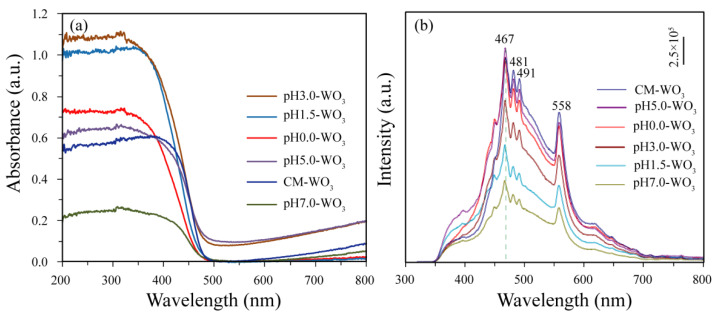
(**a**) UV–vis absorption spectra and (**b**) PL spectra of as-prepared pH*x*-WO_3_ and CM-WO_3_ samples.

The comparison of the UV–vis absorption spectra and PL spectra of the as-prepared pH*x*-WO_3_ and the CM-WO_3_ samples are presented in Figure 8. As demonstrated in Figure 8a, the as-prepared pH*x*-WO_3_ and the CM-WO_3_ samples have similar adsorption spectra, and the absorption edge is at ~475 nm. The light adsorption intensity of the as-prepared pH*x*-WO_3_ and the CM-WO_3_ samples decreases in the order of pH3.0-WO_3_ > pH1.5-WO_3_ > pH0.0-WO_3_ > pH5.0-WO_3_ > CM-WO_3_ > pH7.0-WO_3_. Using the concept of the edge at the intersection of wavelength through extrapolation of the horizontal and sharply rising portions of the curves, the absorption peak for pH0.0-WO_3_, pH1.5-WO_3_, pH3.0-WO_3_, pH5.0-WO_3_, pH7.0-WO_3_, and CM-WO_3_ was determined at 466, 470, 475, 476, 475, and 476 nm, respectively [24,25]. Using the formula *Band gap* = 1240/*Wave length* [24,25], 2.66, 2.64, 2.61, 2.60, 2.61, and 2.60 eV was calculated as band gap energy for pH0.0-WO_3_, pH1.5-WO_3_, pH3.0-WO_3_, pH5.0-WO_3_, pH7.0-WO_3_, and CM-WO_3_ samples, respectively, which are consistent with the energy gap of monoclinic WO_3_ (2.6–2.8 eV), indicating that the pH value had little effect on the band gap of WO_3_ [26]. The photocatalytic activity is related to the charge migration and the recombination rate of photogenerated carriers [9]. Therefore, in order to determine the charge migration and degree of recombination of electron–hole pairs, that is, the separation ability of electron–hole pairs, the photoluminescence (PL) spectra of the as-prepared pH*x*-WO_3_ and the CM-WO_3_ samples was studied as shown in Figure 8b. As shown in Figure 8b, the photoluminescence intensity of the as-prepared pH*x*-WO_3_ and the CM-WO_3_ samples decreases in the order of CM-WO_3_ > pH5.0-WO_3_ > pH0.0-WO_3_ > pH3.0-WO_3_ > pH1.5-WO_3_ > pH7.0-WO_3_. Generally speaking, the recombination rate of electron–hole pairs is related to the intensity of the luminescence peak. The sample with strong luminescence peak intensity indicates the faster recombination rate of electron–hole pairs, whereas the sample with weak luminescence peak intensity indicates the lower recombination rate of electron–hole pairs. Therefore, the high intensity of the luminescence peaks for CM-WO_3_ corresponds to higher charge carrier recombination. Among all the WO_3_ samples, the pH7.0-WO_3_ exhibits the weakest luminescence peak intensity, indicating the lowest charge carrier recombination rate in pH7.0-WO_3_, that is, the pH7.0-WO_3_ offers an excellent photocatalytic activity.

### 3.6. Electrochemical Impedance Spectra Analysis

**Figure 9:**
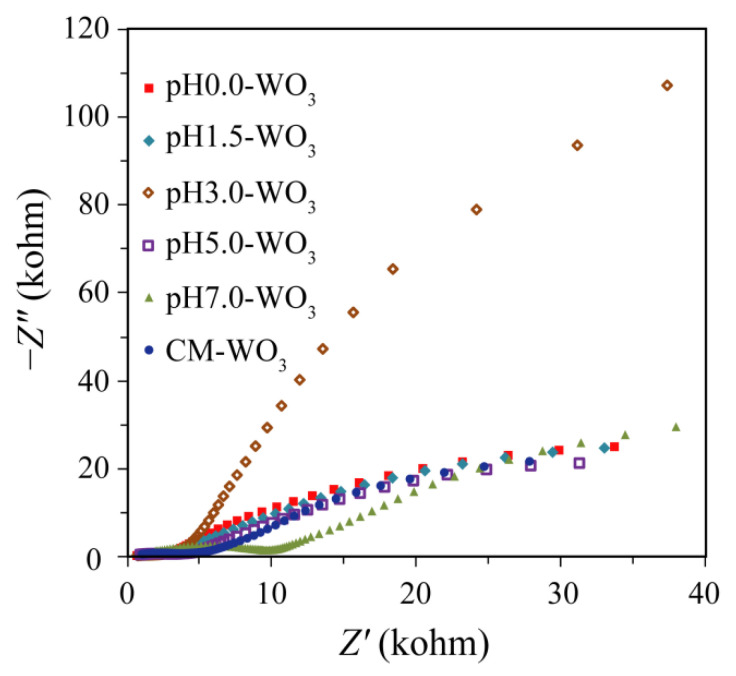
Electrochemical impedance spectra Nyquist plots of the as-prepared pH*x*-WO_3_ and the CM-WO_3_ samples.

Electrochemical impedance spectra (EIS) measurement was employed to investigate the charge migration behavior between the photoinduced electrons and holes [27]. The EIS Nyquist plots of electrodes based on as-prepared pH*x*-WO_3_ and the CM-WO_3_ are shown in Figure 9. The ideal Nyquist plot will show three semicircles in the high-frequency, medium-frequency, and low-frequency ranges, corresponding to the charge transfer at the counter electrode–electrolyte interface, the charge transfer at the oxide–electrolyte interface (*R*_ct_), and the diffusion of ions through the electrolyte, respectively [28]. Under illumination, the photoinduced electron–hole pairs are separated by the applied potential, resulting in the reduction of the *R*_ct_ and the enhancement of the electronic conductivity of the WO_3_ electrode [29]. The diameter of arc radius on the EIS Nyquist plot of the pH7.0-WO_3_ is smaller than that of pH0.0-WO_3_, pH1.5-WO_3_, pH3.0-WO_3_, pH5.0-WO_3_, and CM-WO_3_, indicating that the pH7.0-WO_3_ has the lowest electric resistance and the highest conductivity. As we all know that the rapid separation of photoexcited electron–hole pairs is essential to improving photocatalytic activity [30]. The high conductivity of pH7.0-WO_3_ is also conducive to the transfer of electrons, thus promoting an effective charge separation [30]. The Nyquist plots of pH0.0-WO_3_, pH1.5-WO_3_, pH5.0-WO_3_, and CM-WO_3_ have similar trends, and no typical semicircle is observed at high frequency, indicating the lower electron–hole separation efficiency. The diameter of arc radius on the EIS Nyquist plot of the pH3.0-WO_3_ is bigger than that of other WO_3_, suggesting that the pH3.0-WO_3_ has the highest electric resistance. No typical semicircle is observed on the EIS Nyquist plot of pH3.0-WO_3_, indicating that the charge separation efficiency of pH3.0-WO_3_ is also very low and lower than that of other WO_3_ samples.

### 3.7. Photocatalytic Activity Analysis

**Figure 10:**
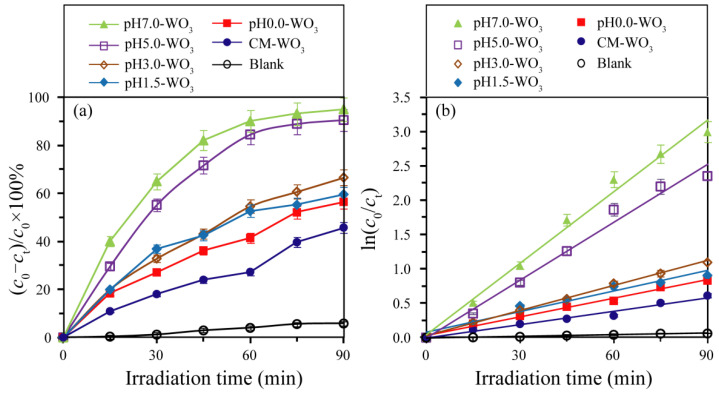
(**a**) Photocatalytic degradation curves of methylene blue solution and (**b**) corresponding kinetic fitting curves.

The photocatalytic activities of WO_3_ samples prepared by adjusting the pH value in the hydrothermal tungstic acid colloidal suspension were evaluated by the decolorization of the MB solution. The adsorption values [mol(MB)/g(WO_3_)] of MB on the surface of WO_3_ samples were 9.57 × 10^−5^, 5.72 × 10^−5^, 1.11 × 10^−4^, 1.13 × 10^−4^, 1.57 × 10^−4^, and 8.88 × 10^−5^ mol/g for the pH0.0-WO_3_, pH1.5-WO_3_, pH3.0-WO_3_, pH5.0-WO_3_, pH7.0-WO_3_, and CM-WO_3_ samples, respectively. These results indicated that the enhancement order of adsorption binding of MB to WO_3_ was pH1.5-WO_3_ < CM-WO_3_ < pH0.0-WO_3_ < pH3.0-WO_3_ < pH5.0-WO_3_ < pH7.0-WO_3_ and that the strong anchoring of MB onto the surface of pH7.0-WO_3_ could improve the photocatalytic activity [31]. As shown in Figure 10a, under UV light irradiation for 90 min, the photodegradation efficiency of MB increased in the order of blank (5.8%) < CM-WO_3_ (45.7%) < pH0.0-WO_3_ (56.4%) < pH1.5-WO_3_ (59.5%) < pH3.0-WO_3_ (66.6%) < pH5.0-WO_3_ (90.5%) < pH7.0-WO_3_ (95.0%). That is, pH7.0-WO_3_ shows the highest photocatalytic activity for the degradation of MB solution, and its degradation efficiency is 1.05, 1.43, 1.60, 1.68, 2.08, and 16.38 times that of pH5.0-WO_3_, pH3.0-WO_3_, pH1.5-WO_3_, pH0.0-WO_3_, CM-WO_3,_ and blank samples, respectively. To quantitatively investigate the photocatalytic reaction kinetics, the experimental data of the MB degradation were fitted by a first-order model as expressed by the formula: ln(*c*_0_/*c*_t_) = *kt*, where *c*_0_/*c*_t_ is the ratio of the concentration dye at adsorption−desorption equilibrium and after various intervals of time and *k* is the apparent first-order rate constant [27]. It can be seen from Figure 10b that there is a linear relationship between ln(*c*_0_/*c*_t_) and *t*, which indicates that the photodegradation reactions follow pseudo-first-order kinetics with apparent rate constants 0.0008, 0.0065, 0.009, 0.0099, 0.0121, 0.0281, and 0.0347 min^−1^ for blank, CM-WO_3_, pH0.0-WO_3_, pH1.5-WO_3_, pH3.0-WO_3_, pH5.0-WO_3,_ and pH7.0-WO_3_, respectively. Obviously, pH7.0-WO_3_ has the largest degradation rate constant, which is 1.23, 2.87, 3.51, 3.86, 5.34, and 43.38 times that of pH5.0-WO_3_, pH3.0-WO_3_, pH1.5-WO_3_, pH0.0-WO_3_, CM-WO_3,_ and blank samples, respectively. All the pH*x*-WO_3_ samples display higher performance in degrading MB than CM-WO_3_. Moreover, for the pH*x*-WO_3_ samples, the photocatalytic performance is enhanced with the increase in pH value of the suspension, and the photocatalytic activity of pH7.0-WO_3_ is the highest, which is 3.86 times that of pH0.0-WO_3_.

It is well known that the photocatalytic activity of photocatalysts is related to the crystalline phase, crystal morphology, grain size, specific surface area, and exposed crystal facets [32]. The crystalline phase of pH*x*-WO_3_ and CM-WO_3_ samples is the same, so the crystalline phase is not the main factor affecting their photocatalytic activities. Based on the previous analysis, it can be seen that the co-exposed crystal facets of pH5.0-WO_3_ ({110} and {002} facets) and pH7.0-WO_3_ ({202} and {020} facets) nanocrystals with irregular morphology are different from those of pH0.0-WO_3_, pH1.5-WO_3_, and pH3.0-WO_3_ nanocrystals ({002}, {020}, and {200} facets) with rod-shaped morphology. Generally speaking, the high-energy {020} and {002} facets can provide more reactive sites, resulting in the enhanced photocatalytic activity of WO_3_ [15]. However, compared with pH0.0-WO_3_, pH1.5-WO_3_, and pH3.0-WO_3_ nanocrystals, the proportion of high-energy {020} and {002} crystal planes in pH5.0-WO_3_ and pH7.0-WO_3_ nanocrystals is less, so the exposure of crystal planes is not the main factor affecting their photocatalytic activities.

The particle size of the crystal is inversely proportional to the specific surface area. Generally speaking, in photochemical reaction, a smaller particle size (enhancing redox capacity) is conducive to the acceleration of the migration rate of photogenerated electrons and holes and the deceleration of the recombination rate [33], and a larger specific surface area (providing more adsorption sites) is conducive to the adsorption of dye molecules on the surface of the catalyst [34], thereby improving the photocatalytic activity of the catalyst. Based on the above discussion, the specific surface area ranks in the order of pH7.0-WO_3_ (14.3 m^2^/g) > pH5.0-WO_3_ (13.7 m^2^/g) > pH3.0-WO_3_ (7.7 m^2^/g) > pH0.0-WO_3_ (6.4 m^2^/g) > pH1.5-WO_3_ (5.4 m^2^/g) > CM-WO_3_ (2.6 m^2^/g). The increasing order of photocatalytic activity is blank < CM-WO_3_ < pH0.0-WO_3_ < pH1.5-WO_3_ < pH3.0-WO_3_ < pH5.0-WO_3_ < pH7.0-WO_3_, which is almost the same as that of specific surface area. Moreover, according to the previous PL and EIS analysis, pH7.0-WO_3_ has the weakest luminescence peak intensity and the smallest arc radius diameter, indicating that the pH7.0-WO_3_ has the highest separation and transfer efficiency and the lowest recombination rate of photoinduced electron–hole pairs.

**Figure 11:**
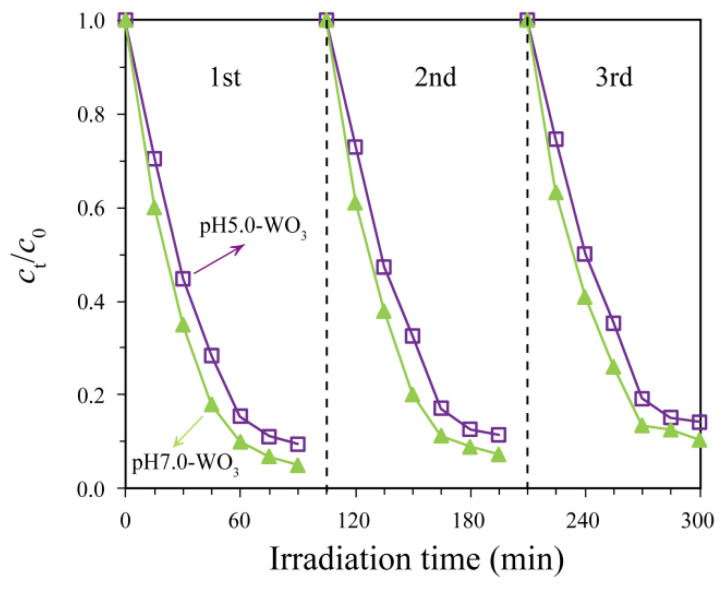
Cyclic degradation curves of pH5.0-WO_3_ and pH7.0-WO_3_ for methylene blue solution.

Multiple use evaluations of a photocatalyst can predict its long-term performance and economic viability. The reusability of pH5.0-WO_3_ and pH7.0-WO_3_ for MB photocatalytic degradation efficiency was examined, as shown in Figure 11. Filtered the methylene blue solution containing solid catalyst after illumination to re-obtain pH5.0-WO_3_ or pH7.0-WO_3_ solid, and then dry it naturally for subsequent use. For pH5.0-WO_3_, 90.5% MB degradation is found in the first run which decreases to 88.5% and 85.8% in the second and third run, respectively. For pH7.0-WO_3_, 95.0% MB degradation is found in the first run which decreases to 92.7% and 89.6% in the second and third run, respectively. The photocatalytic activity of pH5.0-WO_3_ and pH7.0-WO_3_ decreased only 4.7% and 5.4% for MB, respectively, after three consecutive cycles, indicating that the pH5.0-WO_3_ and pH7.0-WO_3_ possessed good reusability.

**Figure 12:**
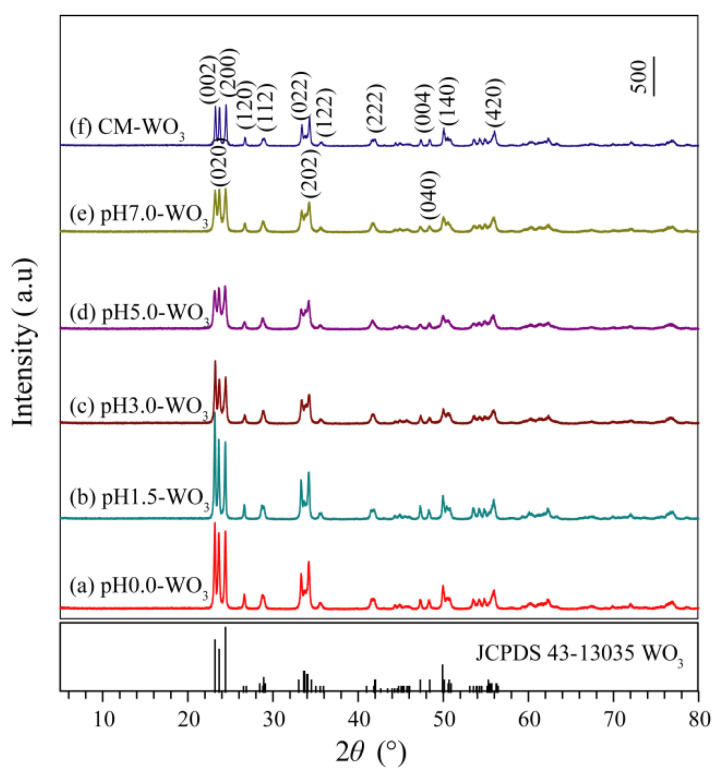
XRD patterns of WO_3_ samples separated from methylene blue solution after photocatalytic degradation: (**a**) pH0.0-WO_3_, (**b**) pH1.5-WO_3_, (**c**) pH3.0-WO_3_, (**d**) pH5.0-WO_3_, (**e**) pH7.0-WO_3_, (f) CM-WO_3_,.

Figure 12 is the XRD patterns of WO_3_ samples separated from methylene blue solution after photocatalytic degradation. It can be seen from Figure 12 that the crystal structure of pH*x*-WO_3_ and CM-WO_3_ has not changed after the photocatalytic reaction, indicating that they are stable during the photocatalytic reaction. Compared with Figure 2, only the crystallinity is reduced, which is caused by the dispersion of the agglomerated particles under vigorous stirring conditions.

## 4. Conclusions

In summary, rod-shaped WO_3_ nanocrystals with co-exposed {002}, {020} and {200} facets, cuboid-shaped WO_3_ nanocrystals with co-exposed {020} and {200} facets, and irregular WO_3_ nanocrystals with co-exposed {110} and {002} facets (or {202) and {020} crystal facets) were synthesized by a simple hydrothermal treatment of white tungstic acid colloidal suspension with desired pH values. The crystal structure, morphology, specific surface area, pore size distribution, chemical composition, electronic states of the elements, optical properties, and charge migration behavior of as-obtained WO_3_ products were characterized by XRD, FESEM, TEM, HRTEM, XPS, fully automatic specific surface area and porosity analyzer, UV–vis absorption spectra, PL spectra, and EIS. Photocatalytic degradation of MB performance of the as-obtained WO_3_ nanocrystals was investigated under ultraviolet irradiation. The increasing order of photocatalytic activity is blank < CM-WO_3_ < pH0.0-WO_3_ < pH1.5-WO_3_ < pH3.0-WO_3_ < pH5.0-WO_3_ < pH7.0-WO_3_. The highest photocatalytic activity of pH7.0-WO_3_ could be attributed to the synergistic effects of the largest specific surface area, the weakest luminescence peak intensity, and the smallest arc radius diameter in comparison with CM-WO_3_ and other pH*x*-WO_3_ nanocrystals.

## Data Availability

Data are contained within the article and are also available from the first corresponding author.
